# Focusing Attention on Muscle Exertion Increases EEG Coherence in an Endurance Cycling Task

**DOI:** 10.3389/fpsyg.2018.01249

**Published:** 2018-07-20

**Authors:** Selenia di Fronso, Gabriella Tamburro, Claudio Robazza, Laura Bortoli, Silvia Comani, Maurizio Bertollo

**Affiliations:** BIND-Behavioral Imaging and Neural Dynamics Center, Department of Medicine and Aging Sciences, “G. d'Annunzio” University of Chieti-Pescara, Chieti, Italy

**Keywords:** functional brain connectivity, focus of attention, MAP model, RPE, effort, performance

## Abstract

The aim of this study was to examine EEG coherence before, during, and after time to exhaustion (TTE) trials in an endurance cycling task, as well as the effect of effort level and attentional focus (i.e., functional external, functional internal, and dysfunctional internal associative strategies−leading to Type 1, Type 2, and Type 3 performances) on brain functional connectivity. Eleven college-aged participants performed the TTE test on a cycle-ergometer with simultaneous EEG and rate of perceived exertion (RPE) monitoring. EEG data from 32 electrodes were divided into five effort level periods based on RPE values (Baseline, RPE 0-4, RPE 5-8, RPE 9-MAX, and Recovery). Within subjects RM-ANOVA was conducted to examine time to task completion across Type 1, Type 2, and Type 3 performance trials. RM-ANOVA (3 performance types × 5 effort levels) was also performed to compare the EEG coherence matrices in the alpha and beta bands for 13 pairs of electrodes (F3-F4, F3-P3, F4-P4, T7-T8, T7-P3, C3-C4, C3-P3, C4-P4, T8-P4, P3-P4, P3-O1, P4-O2, O2-O1). Significant differences were observed on TTE performance outcomes between Type 1 and Type 3, and between Type 2 and Type 3 performance states (*p* < 0.05), whereas Type 1 and Type 2 performance states did not differ. No significant main effects were observed on performance type (*p* > 0.05) for all frequency bands in any pair of electrodes of the coherence matrices. Higher EEG coherence values were observed at rest (Baseline) than during cycling (RPE 0–4, 5–8, 9–MAX) for all pairs of electrodes and EEG frequency bands irrespective of the type of performance (main effect of effort, *p* < 0.05). Interestingly, we observed a performance × effort interaction in C3–C4 in beta 3 band [*F*_(4, 77)_ = 2.62, *p* = 0.038] during RPE 9-MAX for Type 3 performance as compared to Type 1 and Type 2 performances. These findings may have practical implications in the development of performance optimization strategies in cycling, as we found that focusing attention on a core component of the action could stimulate functional connectivity among specific brain areas and lead to enhanced performance.

## Introduction

Physical exercise is commonly known to result in changes of brain cortical activity measured by EEG techniques (Schneider et al., 2009a). It is also generally accepted that exercise can cause temporary changes of the EEG activity in the alpha and beta bands (Crabbe and Dishman, 2004). Specific brain changes are related to different kinds of exercise and to the participants' preferences for physical exercise (Schneider et al., 2009a,b). Moreover, the effects of exercise on brain cortical activity are reflected in specific brain regions. For example, research findings from studies comparing running and cycling revealed an increased alpha activity in the frontal regions, involved in emotional processing, immediately after treadmill exercise on those participants who displayed a clear preference for running. On the other hand, participants who preferred cycling showed increased alpha activity in the parietal regions, which play an important role in integrating sensory information, after bike exercise (Schneider et al., 2009b). These studies have shown the occurrence of brain activity changes as a result of exercise. However, few studies have examined these changes during exercise. For example, Bailey et al. (2008) documented increased EEG power spectra during graded-exercise to fatigue; in particular, they found increased power in the theta, alpha, and beta bands during exercise at different electrode sites (i.e., F3, F4, F7, F8, C3, C4, P3, P4). Findings suggest that brain activity may be related to exercise intensity, which could have an impact on brain waves; in particular, increases in EEG activity in alpha and beta bands seem to reflect augmented ventilatory rate.

In a cycling study, Hottenrott et al. (2013) found cortical activity to be influenced by cadence. Specifically, EEG data indicated that cadence not only directly increased metabolic and cardiac activity, but also influenced cortical parameters. In detail, the U-shaped curve of EEG spectral power over time suggests a central activation that decreases with the onset of fatigue.

Hilty et al. (2011), in addition, demonstrated that there is a fatigue-induced increase in communication between the mid/anterior insular and the motor cortex during cycling exercise. The authors provided basis to further investigate the cortical mechanisms of supraspinal fatigue. Moreover, in different tasks, Babiloni et al. (2011) speculated on the physiological meaning of inter- and intra-hemispheric connectivity during performance; in a golf putting task, for instance, they suggested that the increase of bilateral parietal central coherence in alpha band is due to the recruitment of central-parietal resources related to global attention. However, there is scant research examining brain activity before, during, and after endurance tasks or under stressful conditions.

Interaction among brain processes, cognition, and performance has been recently studied using the multi-action plan (MAP) model (Bortoli et al., 2012; Robazza et al., 2016) as a theoretical framework (for a review, see di Fronso et al., 2017). This model provides practical indications to help athletes reach and maintain optimal performance also under strenuous or stressful situations. The MAP model is based on the notion that different attentional strategies lead to optimal and suboptimal performance states, which are related to specific psychophysiological (Bertollo et al., 2013; Filho et al., 2015), neural (Bertollo et al., 2016; di Fronso et al., 2016), or affective responses (Robazza et al., 2016). The MAP model is conceptualized in function of distinct performance levels (i.e., optimal or suboptimal) and attentional demands (i.e., automatic or controlled). The interplay between performance and attention leads to four performance states: (a) Type 1, optimal-automated performance, characterized by an automatic (“flow” like) attentional mode; (b) Type 2, optimal-controlled performance, typified by an associative focus directed toward core components of a given task/action; (c) Type 3, suboptimal-controlled performance, characterized by a focus directed toward irrelevant information and/or a dysfunctional control of automated action components; and (d) Type 4, sub-optimal-automated performance, typified by a low level or a lack of focus of attention. In particular, former studies showed that athletes can achieve optimal performances (Type 1, Type 2) with different type of effort, attention, and investment of cognitive resources (Furley et al., 2015; Carson and Collins, 2016; Robazza et al., 2016). In brain studies, high levels of anxiety are commonly associated with beta activity increase and alpha activity decrease (e.g., Carvalho et al., 2013). Similarly, efficient and inefficient processing during performance were shown to be modulated by the degree of effort and attentional demands of the task, with a clear Event-Related Synchronization in Type 1 performance and Desynchronization in Type 3 performance (Bertollo et al., 2016).

Drawing on the MAP model assumptions, the effect of different internal and external associative strategies on endurance performance has been investigated (Bertollo et al., 2015). Participants were required to direct attention externally on pacing (Type 1 performance), focus internally on a core component of the cycling action (Type 2), or attend to muscular exertion (Type 3). Findings showed that participants in Type 1 and Type 2 performance states attained optimal performance. On the other hand, Type 3 performance condition led to poor performance because of enhanced feelings of fatigue. Type 4 performance was not considered in this earlier study and in the current investigation because of the difficulty to instate in participants involved in a laboratory task a mental attitude of real disengagement featuring a Type 4 performance state. In a study testing the association between cortical functional networks and the performance types foreseen in the MAP model in cycling (Comani et al., 2014), coherence analysis has been used as an adequate metrics to quantify the functional correlation between active brain areas at the sensor level, as also suggested in more recent studies (Srinivasan et al., 2007; Bowyer, 2016). Comani et al. found that performance types relied on fronto-occipital and inter-hemispheric frontal coherence in the alpha band.

Drawing on the MAP model assumptions, we conducted a counterbalanced repeated measure trial to investigate the effect on cortical coherence (i.e., functional connectivity; Florian et al., 1998; Srinivasan et al., 2007) of internal and external attentional strategies during the different periods (i.e., effort levels) of a time to exhaustion (TTE) cycling task. At Baseline, before the TTE test, we expected to observe in participants a higher functional connectivity in the alpha and beta bands as compared to the periods of TTE execution and Recovery, because of a readiness for task execution that translates in a greater communication within all brain regions (Hypothesis 1-effort level effect). Furthermore, we expected to observe a significant interaction between type of performance and effort level (i.e., Baseline, RPE 0-4, RPE 5-8, RPE 9-MAX, Recovery) on functional connectivity (Hypothesis 2-Performance × effort level interaction). In particular, Type 3 performance should result in a higher level of coherence in the beta band (the biomarker that most reflects motor binding; Cheron et al., 2016), during the last stages of the TTE task, due to the attentional focus on dysfunctional feelings, which increases the communication among sensory motor areas and should differ from Type 1 and Type 2 performances. On the other hand, Type 1 “flow-like” experience should be characterized by reduced functional connectivity, related to cortical inhibition (Klimesch, 1996; Pfurtscheller, 2003; Klimesch et al., 2007) and brain regions deactivation (Knyazev et al., 2011). Because of the limited research on this topic, the current investigation could be considered exploratory in nature. Specifically, alpha band analysis could provide information about global resting state, whereas beta band could offer information about sensory motor integration (beta1), perception-action coupling (beta2), and selective attention related to the motor task (beta3) (Laufs et al., 2003; Donner and Siegel, 2011; Kilavik et al., 2013; Cheron et al., 2016).

## Method

### Participants

We recruited 12 college-aged students. One student discontinued participation from the experiment due to health reasons. Therefore, 11 students (4 women and 7 men, *M*_age_ = 24.29 years, *SD* = 4.91 years) completed the experimental protocol, consisting of five visits to our exercise physiology laboratory. All volunteers participated regularly in different physical activities of low or moderate intensity and some of them were professional cyclists (see Table 1). After being briefed on the general purpose of the study, the participants agreed to participate and signed a written informed consent. The study was conducted in accordance with the declaration of Helsinki and received approval from the local university ethics committee (University “G. d'Annunzio” of Chieti-Pescara) with application ref. n. 10-21/05/2015.

**Table 1:** Mean (M) and Standard Deviations (SD) of Anaerobic Threshold (AT) and Individual Optimal Pedaling Rate (IOPR) obtained with incremental test, and minutes on constant load phase of Time to Exhaustion Test (TTE) in the three types of performance for the participants.

**Participants**	**Age**	**Gender**	**Expertise**	**AT-VO2 (l/min)**	**AT-VO2 (ml/kg/m)**	**AT-VCO2 (l/min)**	**AT-power (Watt)**	**IOPR**	**AT-HR**	**Time to exhaustion (min)**		
										**Type 1**	**Type 2**	**Type 3**
1	24	M	Novice	1.29	20.50	1.28	145	83	140	13	16	15
2	32	M	Amateur	2.04	26.80	2.27	150	100	152	18	16	9
3	33	M	Elite	1.88	24.40	2.83	200	100	138	19	10	10
4	20	F	Novice	1.12	18.70	1.43	85	68	150	16	14	11
5	28	M	Novice	1.69	20.10	1.85	120	78	161	10	15	13
6	33	M	Amateur	3.01	45.50	3.15	225	80	165	14	12	12
7	19	M	Elite	2.27	37.10	2.26	175	70	169	23	31	19
8	25	M	Novice	2.66	30.09	3.21	170	75	159	13	9	8
9	27	F	Novice	1.21	22.80	1.19	90	54	176	11	12	9
10	24	F	Novice	1.15	21.00	1.00	90	60	138	27	26	20
11	21	F	Novice	1.38	25.5	1.48	90	73	186	12	15	10
Mean	24.29			1.79	26.59	1.99	140	76.45	157.63	16	16	12.36
SD	4.91			0.64	8.2	0.8	48.98	14.38	15.84	5.31	6.69	4.05

### Ratings of perceived exertion (RPE)

RPE was measured through the CR-10 Scale (Borg and Borg, 2001) ranging from “0” (no effort) to “•” (maximal sustainable effort). The verbal anchors were: 0 = nothing at all, 0.5 = extremely weak, 1 = very weak, 2 = weak, 3 = moderate, 5 = strong, 7 = very strong, 10 = extremely strong, • = absolute maximum (a score of 11 is assigned to this anchor). No verbal anchors were used for 4, 6, 8, and 9; the use of CR-10 Scale is instrumental in diminishing ceiling effects, and its ratings are linearly related to various physiological parameters such as VO2max, lactate, and heart rate (Borg, 1998).

### Procedure

Similarly to a previous study (Bertollo et al., 2015), five visits to the laboratory were arranged, with inter-visit intervals of 48–72 h in order to permit physiological Recovery of participants. Two qualified researchers collected the data. Data collection occurred in a quiet (no music playing, and no other people allowed in the laboratory) and safe environment to warrant the comfort of the participants. During the first visit, participants received standard instructions about the use of RPE on the CR-10 scale and performed an incremental test to determine their anaerobic threshold (AT) and individual optimal pedaling rate in revolution/minute (rpm). Heart rate, VO_2_, and VCO_2_ were continuously monitored with the Schiller CS 200 system. During the second visit, an EEG was acquired during a TTE test to check the setting and EEG equipment. At the same time, the precision of the estimated AT and the pedaling rate were verified to proceed as accurately as possible with the other three visits to our laboratory that were important for data collection and the subsequent analysis. The time to exhaustion interval is defined as the maximum interval for which the subject can maintain an exercise intensity equal to AT + 5%, and/or after which he/she reaches volitional exhaustion. During the last three visits, participants performed the TTE test on a monark Cyclo-Ergometer (939 E) with simultaneous EEG monitoring. The TTE test was performed adopting a counterbalanced design. During each visit, one of three MAP-based strategies was randomly used during the constant load phase of the protocol:

1. An external associative strategy—focus on an external pacing-metronome set at the individual optimal pedaling rate—leading to Type 1 performance (instructions for participants: “… during the whole constant load phase, a metronome reproducing your optimal pedaling rate will be activated. Please focus your attention on the pacer and follow the pedaling rhythm…”);
2. A functional internal associative strategy—focus on the internal individual optimal pedaling rate (core component)—leading to Type 2 performance (“…during the whole constant load phase maintain your optimal pedaling rate which is estimated to be *n* revolutions per minutes-RPM. Please, focus your attention on your feet to maintain that rhythm…”);
3. A dysfunctional internal associative strategy—focus on muscle exertion (perception of tension, stiffness, fatigue, soreness, etc.) related to pedaling—leading to Type 3 performance (“…during the whole constant load phase, please focus your attention on your muscle exertion…”).

At the end of the task, participants were asked to complete a manipulation check questionnaire using a 10-point frequency scale with anchors 1 (never) and 10 (always). The questionnaire contained one of the following questions: “How often did you focus your attention on the metronome?” (Type 1 performance condition), “How often did you focus your attention on your feet to maintain individual RPM pacing?” (Type 2 performance condition), and “How often did you focus your attention on your muscle exertion?” (Type 3 performance condition). A frequency adherence under 4, which was considered “often enough,” was adopted as an exclusion criterion.

### Incremental test (first visit)

After a warm up (4 min at 75 watt), oxygen uptake (VO_2_) and carbon dioxide production (VCO_2_) were measured using an incremental protocol on a monark cycle ergometer (939 E). Heart rate, VO_2_, and VCO_2_ were continuously monitored using the Schiller C2 200 System, as above mentioned, to obtain all the physiological parameter useful to identify AT. AT was measured using the V-SLOPE method (Wasserman et al., 1994). Pedaling rate was maintained at 70 rpm, and the workload power output, initially set at 75 Watt, was step-wise increased by 25 Watt every 2 min until exhaustion. After the incremental test, participants were given a 20-min rest period. After this period, participants were asked to pedal at AT + 5% for 10 min to identify their individual optimal pedaling rate, while familiarizing themselves with the study procedures.

### TTE test at individual constant load (second to fifth visit)

During the second visit participants familiarized with TTE protocol. After a resting period of 2 min (no movement for Baseline EEG recording) and a warm up period of 4 min on the cycle ergometer at 60% of AT in Watt, participants performed a constant load exhaustive test at individual constant load (AT + 5%) reporting at the same time their RPE, with pedaling rate fixed at their individual optimal pedaling rate. After exhaustion (absolute maximum effort), there was a Recovery period of 4 min at 60% of AT and a further resting period of 2 min without movement. RPE scores were collected 5 s before the end of each minute during the entire protocol. During the following visits to the laboratory (i.e., 3rd, 4th, and 5th), participants were assigned to one of the three experimental conditions, each defined in a random order and occurring on different days. In order to verify the adherence to the experimental assignments, the above-mentioned manipulation check questionnaire was administered.

### EEG recording and pre-processing

Electroencephalographic data were continuously recorded during the whole protocol using the 32 channels EEG ASAlab system with Waveguard cap (Advanced Neuro Technology, Enschede, Netherlands). This system is supplied with shielded wires to make recordings less susceptible to external noise and movements. Sampling frequency was 512 Hz. The ground electrode (AFz) and common average reference were positioned between Fpz and Fz to ensure low impedance values (<10 KΩ). The 32 electrodes were distributed over the scalp according to the 10/5 system (Oostenveld and Praamstra, 2001). The EEG data were band-pass filtered between 0.3 and 40 Hz. Epochs showing instrumental, ocular, and muscular artifacts were detected using the ASA (Advanced Source Analysis) software (Zanow and Knösche, 2004) with the PCA (Principal Components Analysis) method. Data epochs showing residual artifacts were visually identified by two independent experts and excluded from further analysis.

### EEG data analysis

Pre-processed EEG signals were divided based on the TTE test structure and on the RPE scores. We identified 7 periods: Baseline, warm up, RPE 0–4, RPE 5–8, RPE 9–MAX, Recovery, and rest. We retained the Baseline, RPE 0–4, RPE 5–8, RPE 9–MAX, and Recovery periods for further analysis. Unfortunately, rest periods, where participants were requested to stop pedaling, could not be analyzed because of sweating artifacts due to the autonomic nervous system response, which irremediably affected EEG signals. Warm up, instead, was not a period of interest because attention manipulation was not implemented. For each period we analyzed the EEG intervals of 4 s duration from −6 to −2 s prior to RPE evaluation. Four-seconds epochs were averaged at individual level when part of similar categories of RPE periods (e.g., participant 1: Baseline = 2 epochs for each type of performance; RPE 0–4 = 5 epochs for Type 1; 6 for Type 2; 7 for Type 3; RPE5–8 = 4 epochs for Type 1; 6 for Type 2; 4 for Type3; RPE 9–MAX = 4 epochs for Type 1; 3 for type 2; 4 for Type 3; Recovery = 4 epochs for each type of performance). Therefore, the grand average of the eleven participants was performed resulting in a total of 22 epochs for Baseline; 44 epochs for Recovery, 53 (±10 epochs) for RPE 0–4, 50 (±2) for RPE5–8 44 (±6) for RPE 9-MAX.

Coherence analysis of the EEG data was performed to detect cortical connectivity patterns in relation to the different attentional strategies and time periods. The complex coherence between two signals X_1_ and X_2_ (recorded by two given electrodes) was calculated as the cross-spectrum between the signals and normalized by the square root of the power spectrum product of the two signals. Given that coherence is a normalized measure of the correlation between two signals, and consists of complex values, its amplitude can vary from 0 to 1. For each period, mean coherence matrices were calculated in the alpha (8–12 Hz), beta 1 (12–18 Hz), beta 2 (18–23 Hz), and beta 3 (23–30 Hz) bands (ASA-Lab software). We divided the beta band in three sub-bands to better study sensory motor processes, perception-action coupling, and selective attention (Laufs et al., 2003; Donner and Siegel, 2011; Kilavik et al., 2013; Cheron et al., 2016).

For each of the four frequency bands (i.e., alpha, beta1, beta2, beta3) we obtained 15 reference coherence matrices (3 performance types × 5 time periods) by averaging the mean coherence matrices of the 11 participants. To retain only significant functional connections across EEG signals, we adopted the approach proposed by Berchicci et al. (2015) and thresholded each reference coherence matrix on the basis of its own coherence value distribution, which is expected to be non-Gaussian. We then calculated the Median and Median Absolute Deviation (MAD) of the coherence value distribution for each reference coherence matrix, and defined a new thresholded coherence matrix where only the coherence values > (Median + 1 MAD) were considered as meaningful functional connections and retained, whereas all other coherence values were set equal to zero (Li et al., 2015; Chella et al., 2016). Each thresholded coherence matrix is composed of the coherence values of each pair of 30 electrodes with this order of labels: Fp1, Fpz, Fp2, F7, F3, Fz, F4, F8, FC5, FC1, FC2, FC6, T7, C3, Cz, C4, T8, CP5, CP1, CP2, CP6, P7, P3, Pz, P4, P8, POz, O1, OZ, O2.

### Statistical analysis

Before considering cortical data, we analyzed performance outcomes in TTE test, running a within-subjects repeated measure analysis comparing the time to complete the task during each type of performance (Type 1, Type 2, Type 3).

Afterwards, a series of within-subjects repeated measures ANOVA was performed with 3 (performance types) × 5 (effort level = Baseline, RPE_0–4, RPE_5–8, RPE_9–Max, Recovery) on each pair of electrodes. According to a previous study (Del Percio et al., 2011), representative electrodes are F3, F4, C3, C4, P3, P4, T7, T8, O1, O2. We analyzed cortical activity by combining these electrodes in pairs representative of inter-hemispheric (F3–F4, C3–C4, T7–T8, P3–P4, O2–O1) and intra-hemispheric (F3–P3, F4–P4, T7–P3, T8–P4, C3–P3, C4–P4, P3–O1, P4–O2) connectivity across the frontal, central, parietal, temporal, and occipital regions in alpha and beta bands.

## Results

### Performance outcomes comparison

Within-subjects RM-ANOVA for TTE (see Table 1) showed significant differences among performance outcomes, *F*_(2, 9)_ = 7.323, *p* = 0.013, $\eta_{\text{p}}^{2}$ = 0.619, Power = 0.827*. Post-hoc* pairwise comparisons with Bonferroni correction, showed significant differences between Type 1 and Type 3 (*p* = 0.037) performance states, and between Type 2 and Type 3 (*p* = 0.024), but not between Type 1 and Type 2 (*p* = 1.000).

### Manipulation check

Manipulation check results showed that participants adhered satisfactorily to the experimental conditions. During Type 1 performance condition, response ratings ranged from 4 to 9, which corresponded to an adherence frequency from “often enough” to “almost always” (*M* = 6.80, SD = 1.83). In Type 2 performance state, the response ratings ranged from 5 to 10 (“often” to “always”; M = 7.36, SD = 1.80), whereas in Type 3 performance state the response ratings ranged from 4 to 10 (“often enough” to “always,” *M* = 7.27, *SD* = 1.79).

### EEG coherence results

**Figure 1:**
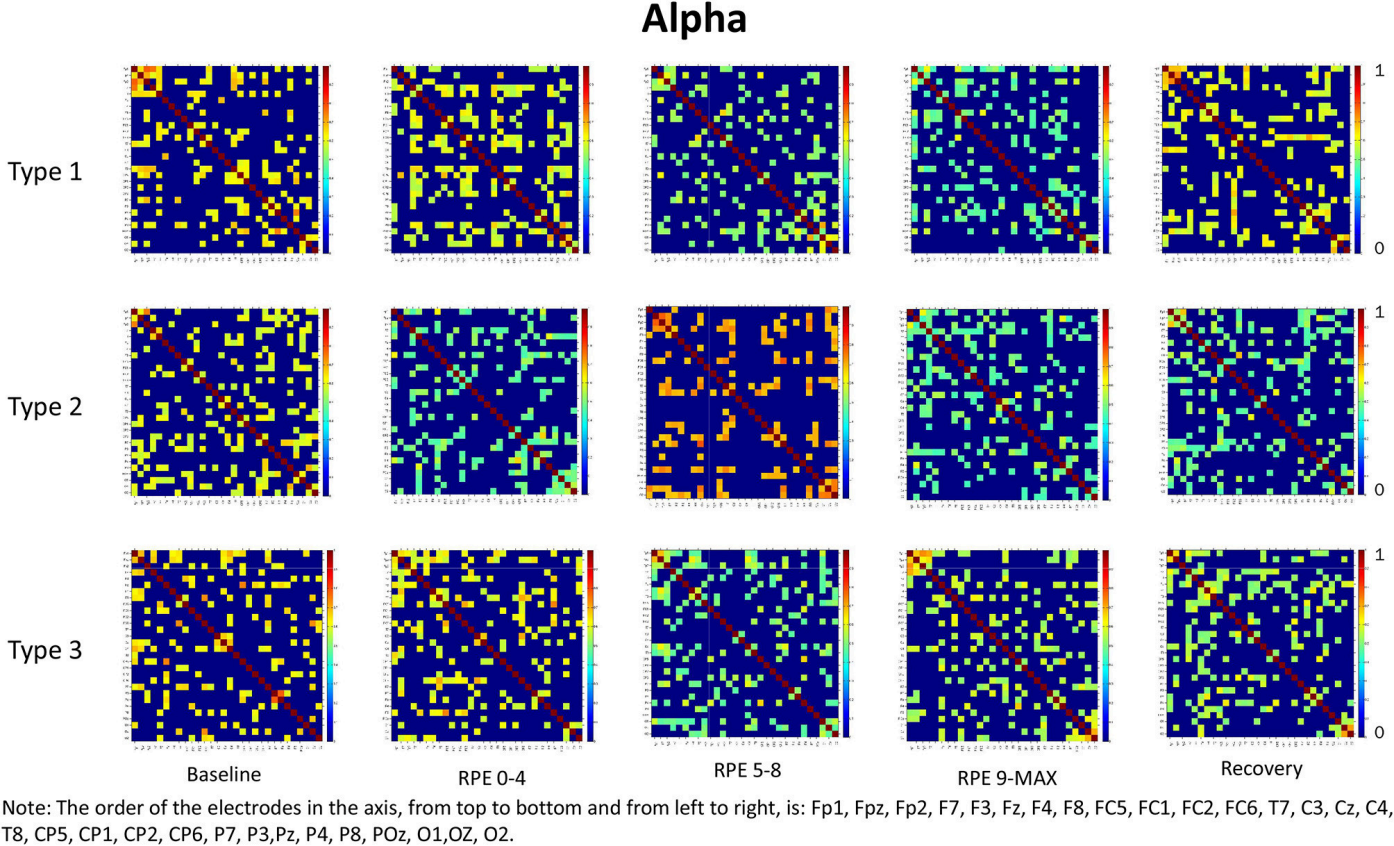
Coherence matrices for each type of performance during different time periods of the protocol in the alpha band. Red color indicates high values of coherence whereas the blue color indicates low values of coherence.

**Figure 2:**
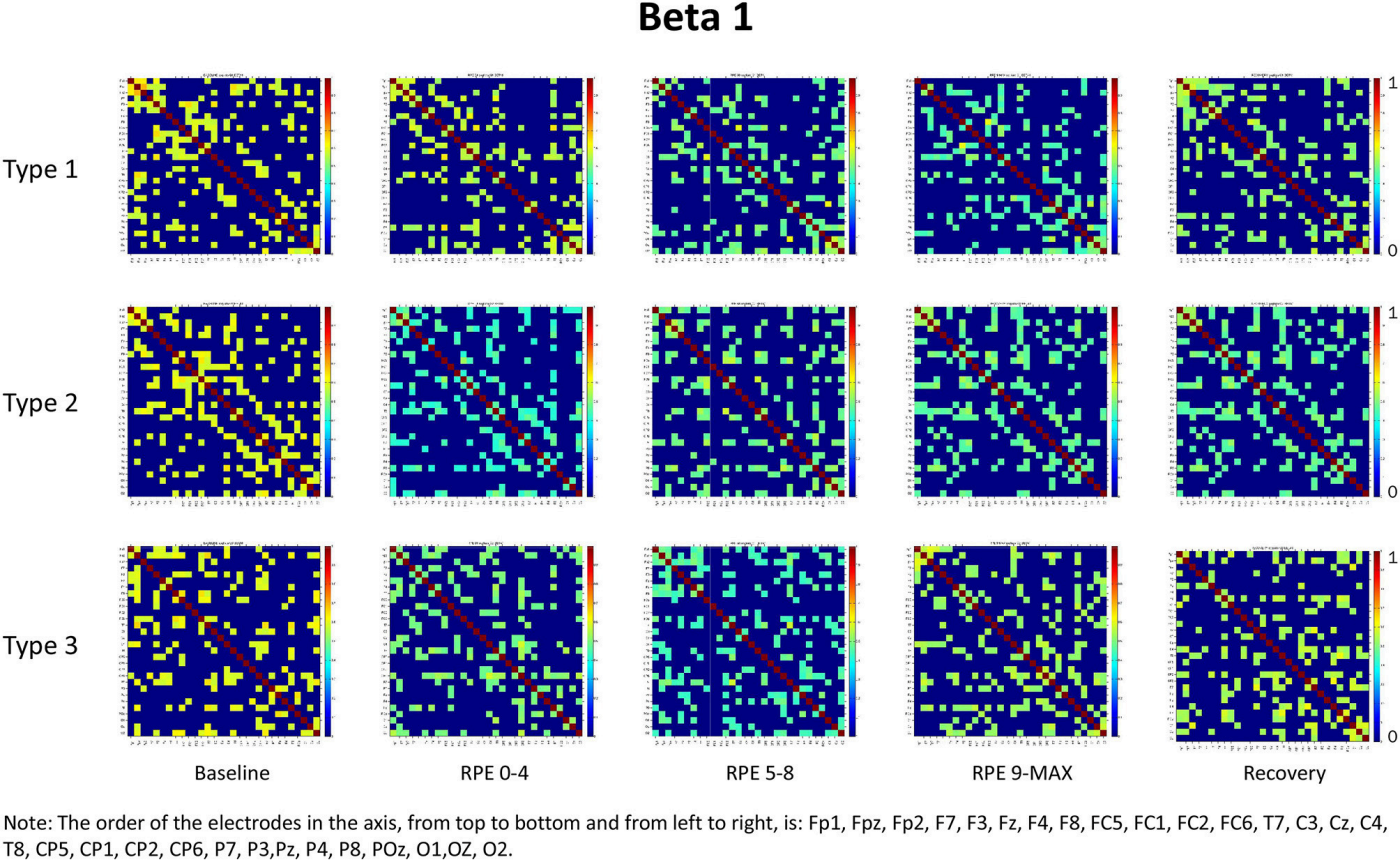
Coherence matrices for each type of performance during different time periods of the protocol in the beta 1 band. Red color indicates high values of coherence whereas the blue color indicates low values of coherence.

**Figure 3:**
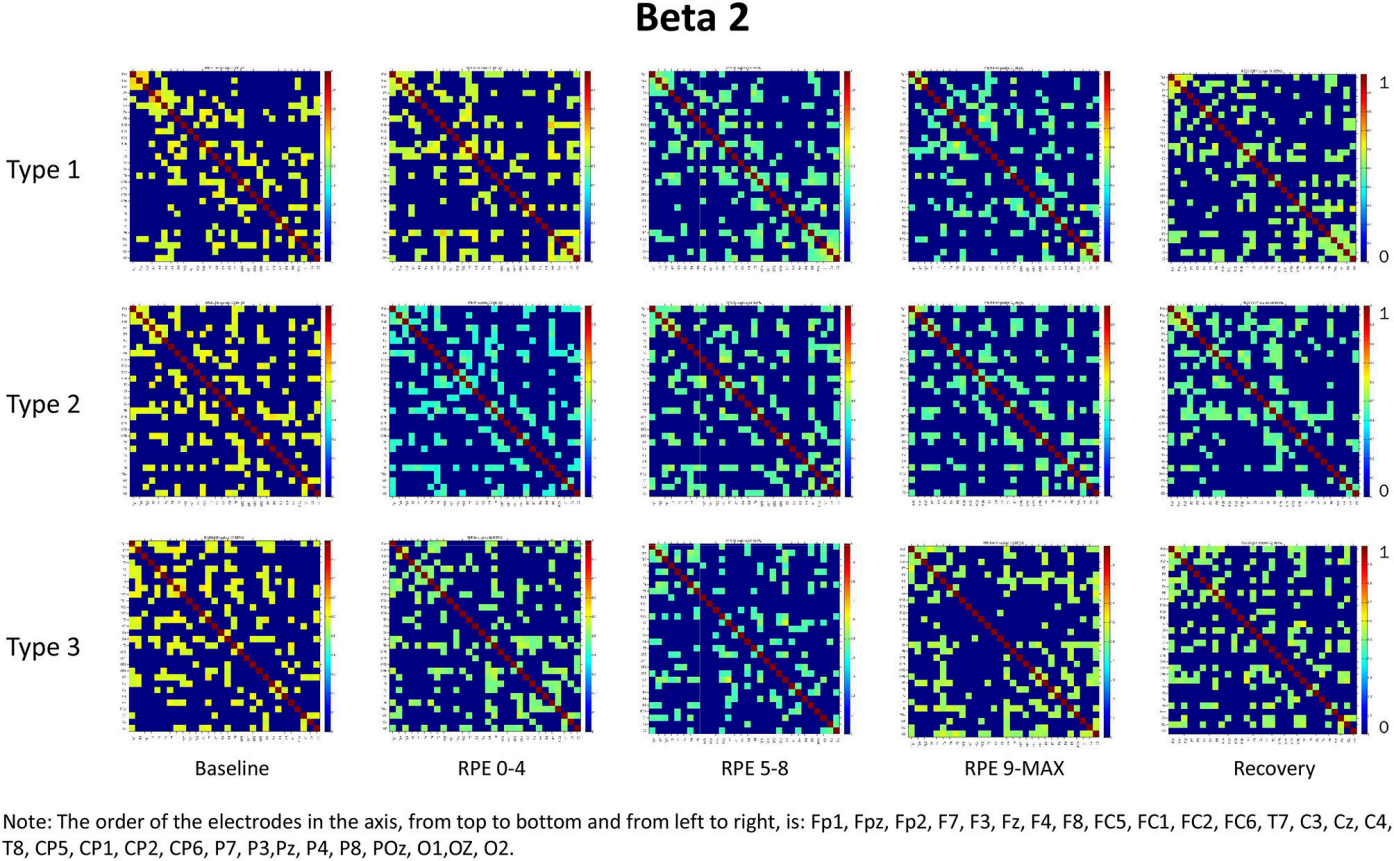
Coherence matrices for each type of performance during different time periods of the protocol in the beta 2 band. Red color indicates high values of coherence whereas the blue color indicates low values of coherence.

**Figure 4:**
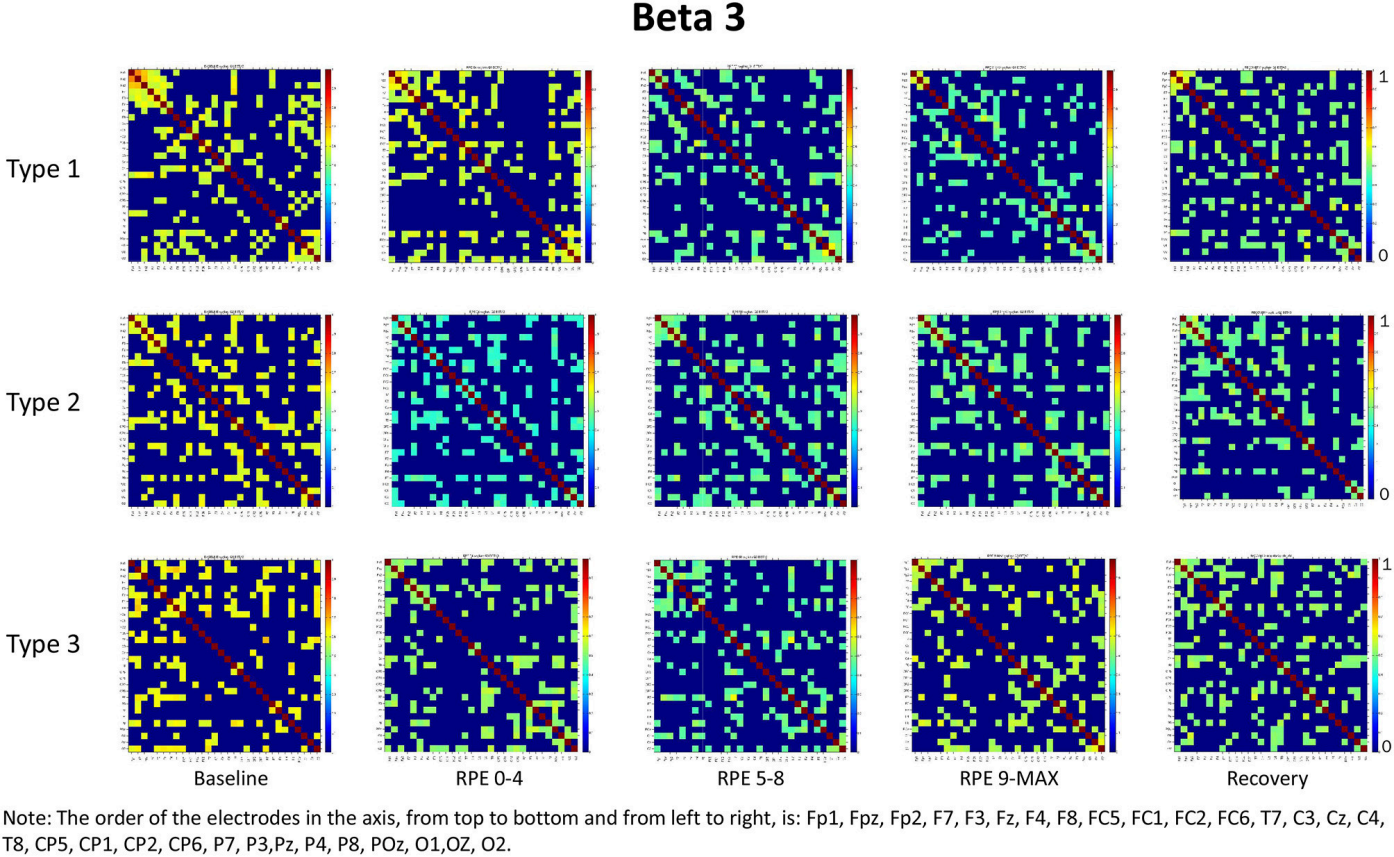
Coherence matrices for each type of performance during different time periods of the protocol in the beta 3 band. Red color indicates high values of coherence whereas the blue color indicates low values of coherence.

For each frequency band (alpha, beta1, beta2, beta3) we calculated coherence matrices for each type of performance (Type 1, Type 2, Type 3) and for each effort level (Baseline, RPE 0–4; RPE 5–8; RPE 9–MAX, Recovery). These matrices are shown in Figures 1–4. Furthermore, the averaged coherence values of all electrodes in the alpha and beta bands are included in Supplementary Materials to provide a comprehensive picture of brain activity at the sensor level (see Figures 1S−4S).

### Alpha band

RM-ANOVA 3 (performance) × 5 (effort level) showed significant effects on effort level for almost all pairs of chosen electrodes (see Table 1S). *Post-hoc* analysis showed differences between Baseline and periods of TTE task (effort level), mainly between Baseline and RPE 5–8 and between Baseline and 9-MAX period (see Table 2).

**Table 2:** *Post-hoc* pairwise comparisons on effort level in alpha band.

**Elec Pair**	**Baseline mean coherence values**	**Other periods mean coherence values**	***P***
F3-P3	0.542	RPE 5–8, 0.422	0.034
F4-P4	0.551	RPE 5–8, 0.397	0.000
T7-T8	0.576	RPE 5–8, 0.460	0.041
	0.576	RPE 9–MAX, 0.475	0.042
T7-P3	0.545	RPE 5–8, 0.406	0.000
	0.545	RPE 9-MAX, 0.400	0.032
C3-C4	0.549	RPE 5–8, 0.414	0.011
C3-P3	0.551	RPE 5–8, 0.442	0.001
	0.551	Recovery, 0.453	0.012
C4-P4	0.570	RPE 5–8, 0.427	0.012
	0.570	RPE 9–MAX, 0.439	0.003
T8-P4	0.540	RPE 5–8, 0.420	0.003
P3-P4	0.580	RPE 5–8, 0.425	0.003
	0.580	Recovery, 0.483	0.039
P4-O2	0.573	RPE0–4, 0.454	0.021
	0.573	RPE5–8, 0.449	0.016
	0.573	RPE 9-MAX, 0.446	0.003
	0.573	Recovery, 0.441	0.022

*Only significant results are reported*.

### Beta bands

RM-ANOVA 3 (performance) × 5 (effort level) showed significant effort level effects for almost all pairs of chosen electrodes in all beta bands (see Tables 2S−4S). *Post-hoc* comparisons on effort level showed differences mainly between Baseline and RPE 5–8, and between Baseline and Recovery (Table 3). Moreover, performance × time interaction was found for C3–C4 electrodes pair in beta 3 band (Table 4S). *Post-hoc* pairwise comparisons showed significant differences between Type 3 and Type 1 performances, as well as between Type 3 and Type 2 performances, but not between Type 1 and Type 2 during RPE 9-MAX (Table 4).

**Table 3:** *Post-hoc* pairwise comparisons on effort level in beta bands.

**Bands**	**Elec Pair**	**Baseline mean coherence values**	**Other periods mean coherence values**	***P***
Beta 1	F3-F4	0.542	Recovery, 0.443	0.001
	F3-P3	0.516	RPE 5–8, 0.370	0.009
	T7-P3	0.502	RPE 5–8, 0.414	0.046
	C3-P3	0.548	Recovery, 0.465	0.028
	C4-P4	0.546	RPE 5–8, 0.421	0.003
		0.546	RPE 9–MAX, 0.465	0.005
	P3-P4	0.559	RPE 5–8, 0.438	0.001
		0.559	Recovery, 0.458	0.045
	P4-O2	0.532	RPE 5– 8, 0.437	0.046
		0.532	RPE9–MAX, 0.413	0.030
		0.532	Recovery, 0.428	0.027
	O2-O1	0.591	RPE 0–4, 0.473	0.040
Beta 2	F3-F4	0.565	RPE 5–8, 0.416	0.029
		0.565	Recovery, 0.444	0.001
	F3-P3	0.539	RPE 5–8, 0.391	0.025
		0.539	Recovery, 0.416	0.047
	F4-P4	0.514	RPE 5– 8, 0.365	0.001
	C3-C4	0.572	RPE 5–8, 0.441	0.005
	C3-P3	0.555	RPE 5–8, 0.433	0.000
		0.555	RPE 9–MAX, 0.448	0.043
		0.555	Recovery, 0.447	0.049
	C4-P4	0.523	RPE 5–8, 0.400	0.005
	T8-P4	0.503	RPE 5– 8, 0.383	0.005
	P3-P4	0.523	RPE 5–8, 0.420	0.002
		0.523	Recovery, 0.428	0.013
	P3-O1	0.508	RPE 5–8, 0.422	0.046
		0.508	Recovery, 0.394	0.036
	P4-O2	0.524	Recovery, 0.430	0.006
Beta 3	F3-F4	0.549	RPE 5–8, 0.411	0.019
		0.549	Recovery, 0.438	0.002
	F3-P3	0.560	RPE 5–8, 0.388	0.000
		0.560	RPE 9–MAX, 0.419	0.002
		0.560	Recovery, 0.420	0.000
	T7-T8	0.555	RPE 5–8, 0.425	0.044
	T7-P3	0.526	RPE 5–8, 0.415	0.003
	C3-C4	0.563	RPE 5–8, 0.415	0.010
	C3-P3	0.538	RPE 5–8, 0.424	0.029
		0.538	Recovery, 0.459	0.000
	C4-P4	0.535	RPE 5–8, 0.408	0.010
		0.535	RPE 9–MAX, 0.449	0.030
		0.535	Recovery, 0.436	0.026
	T8-P4	0.531	RPE 5–8, 0.406	0.000
		0.531	RPE 9–MAX, 0.445	0.050
	P4-O2	0.543	RPE 5–8, 0.435	0.008
		0.543	Recovery, 0.430	0.001
	O2-O1	0.610	Recovery, 0.496	0.026

*Only significant results are reported*.

**Table 4:** *Post-hoc* pairwise comparisons for C3-C4 electrodes pair on performance × effort level interaction (red color indicates *p* < 0.05).

	**T1 Baseline**	**T1 RPE 0–4**	**T1 RPE 5–8**	**T1 RPE 9–MAX**	**T1 Recovery**	**T2 Baseline**	**T2 RPE 0–4**	**T2 RPE 5–8**	**T2 RPE 9–MAX**	**T2 Recovery**	**T3 Baseline**	**T3 RPE 0–4**	**T3 RPE 5–8**	**T3 RPE 9–MAX**	**T3 Recovery**
	**Mean = 0.57**	**Mean = 0.56**	**Mean = 0.41**	**Mean = 0.43**	**Mean = 0.49**	**Mean = 0.57**	**Mean = 0.39**	**Mean = 0.42**	**Mean = 0.41**	**Mean = 0.47**	**Mean = 0.54**	**Mean = 0.47**	**Mean = 0.40**	**Mean = 0.55**	**Mean = 0.48**
**Effort levels (periods)**	***P***	***P***	***P***	***P***	***P***	***P***	***P***	***P***	***P***	***P***	***P***	***P***	***P***	***P***	***P***
Baseline		0.97	0.00	0.01	0.10	0.86	0.00	0.00	0.00	0.05	0.55	0.05	0.00	0.76	0.07
RPE 0–4	0.97		0.00	0.01	0.11	0.83	0.00	0.00	0.00	0.06	0.57	0.05	0.00	0.80	0.08
RPE 5–8	0.00	0.00		0.68	0.13	0.00	0.59	0.85	0.88	0.22	0.01	0.24	0.73	0.01	0.18
RPE 9–MAX	0.01	0.01	0.68		0.27	0.00	0.34	0.82	0.57	0.42	0.03	0.44	0.45	0.02	0.35
Recovery	0.10	0.11	0.13	0.27		0.07	0.04	0.18	0.09	0.76	0.30	0.73	0.06	0.18	0.86
Baseline	0.86	0.83	0.00	0.00	0.07		0.00	0.00	0.00	0.04	0.44	0.03	0.00	0.64	0.05
RPE 0–4	0.00	0.00	0.59	0.34	0.04	0.00		0.47	0.70	0.08	0.00	0.09	0.85	0.00	0.06
RPE 5–8	0.00	0.00	0.85	0.82	0.18	0.00	0.47		0.73	0.30	0.02	0.32	0.59	0.01	0.24
RPE 9–MAX	0.00	0.00	0.88	0.57	0.09	0.00	0.70	0.73		0.17	0.01	0.18	0.85	0.00	0.13
Recovery	0.05	0.06	0.22	0.42	0.76	0.04	0.08	0.30	0.17		0.18	0.97	0.12	0.10	0.90
Baseline	0.55	0.57	0.01	0.03	0.30	0.44	0.00	0.02	0.01	0.18		0.17	0.00	0.76	0.23
RPE 0–4	0.05	0.05	0.24	0.44	0.73	0.03	0.09	0.32	0.18	0.97	0.17		0.13	0.09	0.87
RPE 5–8	0.00	0.00	0.73	0.45	0.06	0.00	0.85	0.59	0.85	0.12	0.00	0.13		0.00	0.09
RPE 9–MAX	0.76	0.80	0.01	0.02	0.18	0.64	0.00	0.01	0.00	0.10	0.76	0.09	0.00		0.13
Recovery	0.07	0.08	0.18	0.35	0.86	0.05	0.06	0.24	0.13	0.90	0.23	0.87	0.09	0.13	

## Discussion

Current results, derived from EEG coherence analysis of data collected before, during, and after TTE, corroborated previous behavioral findings (Bertollo et al., 2015) and helped clarifying the brain mechanisms underpinning endurance task performance. Specifically, results related to performance outcomes confirmed that participants were able to optimally perform using an external associative strategy by focusing their attention on the metronome (Type 1 performance). Similarly, participants were able to achieve optimal performance also using an internal associative strategy with a focus of attention directed to the core component of the action (Type 2 performance). On the other hand, participants' performance was poor when they focused their attention on muscle exertion. These results suggest that individuals may perform well not only in a flow-like performance state (Type 1), but also when they pay attention to specific action components (Type 2 state). Therefore, identifying the core components of action linked to functional performance patterns and focusing attention on this fundamental movement can improve performance and help individuals counteract the discomfort and pain deriving from muscle soreness and fatigue in endurance tasks.

The main purpose of this study was to examine, within the theoretical framework of the MAP model, the effect of attentional strategies on brain functional connectivity during different periods of effort. In summary, findings from the entire scalp (averaged coherence of all electrodes) in the alpha and beta bands showed:

1. Higher coherence values at rest (Baseline) than during task execution (RPE 0–4, 5–8, 9–MAX periods) for all performance types (Hypothesis 1-effort level effect). *Post-hoc* pairwise comparisons showed significant differences between Baseline and the two periods of high effort (RPE 5–8; RPE 9–MAX) in both alpha and beta bands. Differences between Baseline and Recovery were found especially in the beta bands.
2. Higher coherence values in Type 3 performance as compared to Type 1 and Type 2 performances during RPE 9-MAX (Hypothesis 2-Performance × effort level interaction). It is interesting to observe that performance × effort level interaction on the C3–C4 electrodes pair in beta 3 band most likely reflected the effect of focusing attention on muscle exertion (Type 3 performance state), that led to higher inter-hemispheric communication between motor areas during the exhausting phase (i.e., RPE 9-Max).

These results are consistent with previous findings which associate high values of coherence with functional coupling (Thatcher et al., 1986), information exchange (Petsche et al., 1997; Pfurtscheller and Andrew, 1999), and functional coordination (Gevins et al., 1989) among brain regions. High coherence values suggest an increased functional synchronization among two or more brain areas related to the preparation for task execution (Pfurtscheller and Andrew, 1999).

Our findings also highlighted that coherence was generally lower during those periods characterized by movement and high effort such as at RPE 5–8 and particularly at RPE 9–MAX (especially in Type 1 and Type 2 performance).

Our results are partially consistent with the finding that after a fatiguing cycling exercise there is an increase in the communication between the mid/anterior insula and the motor cortex (Hilty et al., 2011), similar to what observed for the Baseline period. Indeed, from a statistical point of view, we found differences for inter- and intra-hemispheric coherence when comparing Baseline and TTE, but not when comparing TTE and Recovery. This occurred not only in the alpha but also in the beta band, the biomarker that best reflects motor binding (Cheron et al., 2016). Therefore, we can argue that our first hypothesis was confirmed to a large extent, although further research is necessary to compare TTE and Recovery immediately after exercise, eventually considering longer periods of post-exercise Recovery.

We also noticed that coherence patterns during Baseline periods were similar for all types of performance. This finding indicates an extensive exchange of background information among all brain regions before task execution and movement (Petsche et al., 1997; Pfurtscheller and Andrew, 1999). Even if, in general, we did not observe significant results on performances by effort interactions, it is worth noting that Type 1 performance was usually typified by higher coherence as compared to the other performance types during low effort (RPE 0–4 period) in the alpha and beta bands. This result could be related to the external associative strategy adopted that can initially engage a broader functional connectivity across brain areas, as well as to the nature of the task that requires a sustained movement (Comani et al., 2014). Lower coherence in Type 2 and Type 3 performances could be related to negative feelings associated with fatigue or the internal focus of attention (Wilson et al., 2011). Of note, during maximum effort (RPE 9–MAX period) Type 1 performance was characterized by lower coherence that could be due to fatigue effects. These may lead to a reorganization of the brain network (Berchicci et al., 2013) irrespective of the functional strategy adopted. Moreover, we can hypothesize that task execution during the final period of the TTE becomes more automatic and needs less information exchange, especially when an external associative strategy is adopted (Hatfield and Kerick, 2007). This result can be also partially interpreted within the framework of the neural efficiency hypothesis, which reflects a general reduction in neural activity as task execution becomes more automated and less controlled (Callan and Naito, 2014). We also found that an external associative strategy was effective and possibly leading to flow like experiences, as demonstrated by cortical inhibition and therefore by the low coherence values obtained during maximum effort (Knyazev et al., 2011).

It is worth considering that during the Recovery phase there was no attention manipulation and, consequently, cognitive and attentional demands were low; these low demands could have contributed to the retrieval of coherence patterns similar to those observed during Baseline. From the visual inspection of the coherence matrices, indeed, we observed, especially in the alpha band, high coherence values during Recovery in Type 1 performance, similarly to coherence in Type 3 performance. We can hypothesize that after maximum effort a counteracting mechanism restores the same pattern of functional connectivity among brain areas as during Baseline. Indeed sensory-related thalamic nuclei information, which serves as gate to the primary sensory areas in the cortex during TTE (Beiser and Houk, 1998), is absent during Baseline. We also observed another difference between Type 1 and Type 3 performance states: coherence values in beta 3 were significantly higher for Type 3 during maximum effort in C3–C4. This may be due to the focus of attention on muscle exertion that leads to an increased inter-hemispheric communication between motor areas devoted to the motor input in the bilateral cycling task (Comani et al., 2014). We could argue that an enhancement of alpha and beta interhemispheric coherence, particularly in C3–C4, may reflect the primary activation of the somatosensory cortex in noxious processing (Chen and Rappelsberger, 1994). This difference was also observed between Type 2 and Type 3 performance, but not between Type 1 and Type 2 performance. These findings corroborate the MAP model perspective because the two optimal performances (Type 1 and 2) showed similar functional connectivity patterns, which were different from those observed for Type 3 performance (Bertollo et al., 2016; di Fronso et al., 2016, 2017). However, our findings are not conclusive in regard to our second hypothesis, and further investigation is needed to more deeply explore sensory motor integration and perception-action coupling. From an applied perspective, this study suggests the use of bio-neurofeedback not just to help people divert their attention away from dysfunctional sensations (Bertollo et al., 2015), but also to stimulate functional connectivity among specific brain areas for performance optimization, according to the explanation provided by Poldrack (2015) on neural efficiency theory. He suggested that “the total amount of energy consumed by neuronal computations depends not just upon the function of individual neurons, but also on how those neurons are connected to one another” (p. 16). Neurofeedback training could be used together with relaxation and mental skills training to help athletes modulate alpha or beta brain waves, and therefore self-regulate their functional arousal level (di Fronso et al., 2017).

Some limitations of the current study need to be addressed in future research. From a methodological point of view, performing EEG studies in sport is still hard due to different types of artifacts affecting the EEG recordings. We conducted this study with a stationary EEG system and wet electrodes, which did not totally allow to avoid the artifacts in low frequency ranges. More advanced EEG equipment (e.g., mobile EEG systems, dry-electrode technology) and new algorithms for EEG data pre-processing (Stone et al., 2018; Tamburro et al., 2018) could allow to consider also the theta band, hence paving the way to a better interpretation of EEG data in the light of the neural efficiency hypothesis. From a theoretical point of view, it might be important to investigate also cortico-muscolar coherence during voluntary movements (Marsden et al., 2000), as it could provide useful information on specific functional connections between the cortex and the engaged muscles (Travis et al., 2002), and a better understanding of the brain-body interaction and integration (Tang and Bruya, 2017). Finally, future studies should engage a larger number of participants to provide more reliable results, especially about peak performance experiences that are rare to find and difficult to reproduce. Research should also be extended to different endurance sports and more experienced athletes to attain more generalizable findings. Other functional neuroimaging techniques (e.g., NIRS) and analytic procedures (i.e., LORETA) could also enable a better understanding of structure-function and brain-body connections.

## Author contributions

All authors listed have made a substantial, direct and intellectual contribution to the work, and approved it for publication.

### Conflict of interest statement

The authors declare that the research was conducted in the absence of any commercial or financial relationships that could be construed as a potential conflict of interest.
