# Supplementary material for: Focusing Attention on Muscle Exertion Increases EEG Coherence in an Endurance Cycling Task
Source: Front Psychol. 2018 Jul 20;9:1249. doi: 10.3389/fpsyg.2018.01249 (PMC6063037; doi:10.3389/fpsyg.2018.01249)
Supplement: Supplementary file 1 [file Data_Sheet_1.docx]

Supplementary Material

Figure 1S. Averaged coherence values (not tresholded) of the 10 selected electrodes (F3, F4, C3, C4, P3, P4, T7, T8, O1, O2) for each Type of performance during each effort period in alpha band


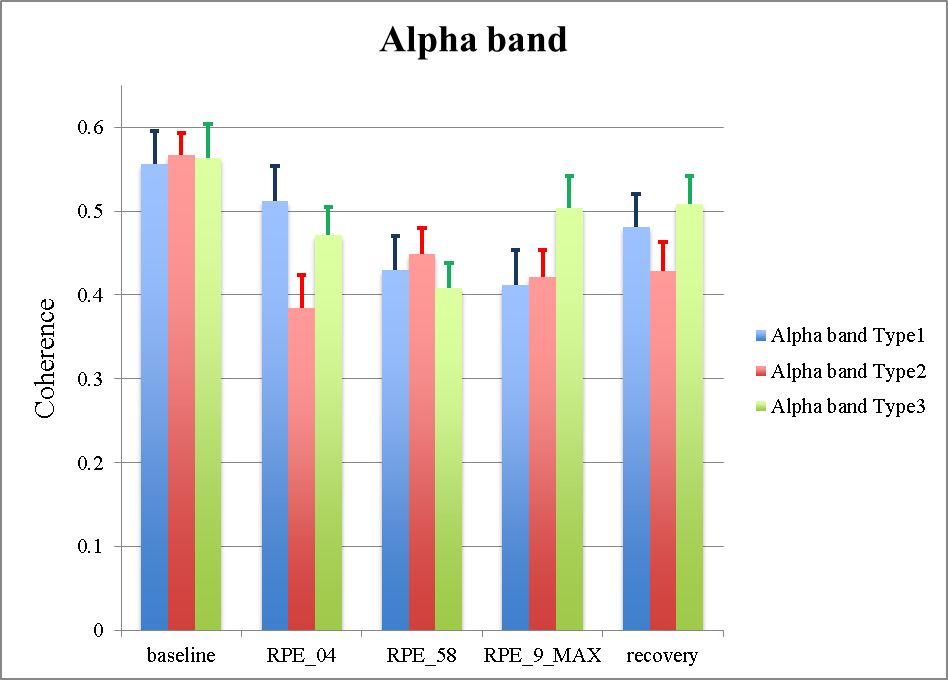


Figure 2S. Averaged coherence values (not tresholded) of the 10 selected electrodes (F3, F4, C3, C4, P3, P4, T7, T8, O1, O2) for each Type of performance during each effort period in beta 1 band


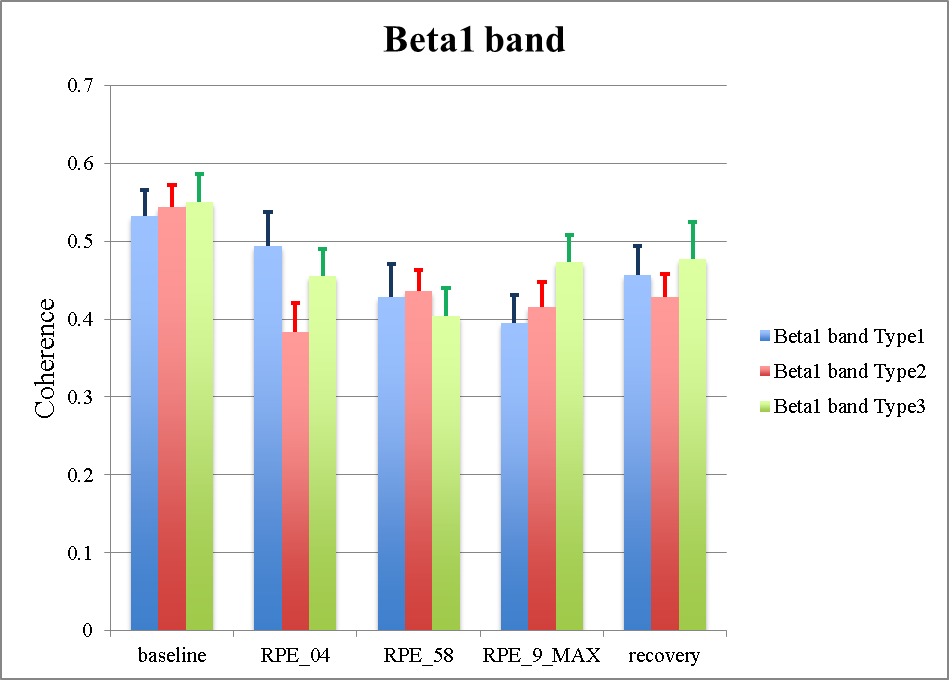


Figure 3S. Averaged coherence values (not tresholded) of the 10 selected electrodes (F3, F4, C3, C4, P3, P4, T7, T8, O1, O2) for each Type of performance during each effort period in beta 2 band


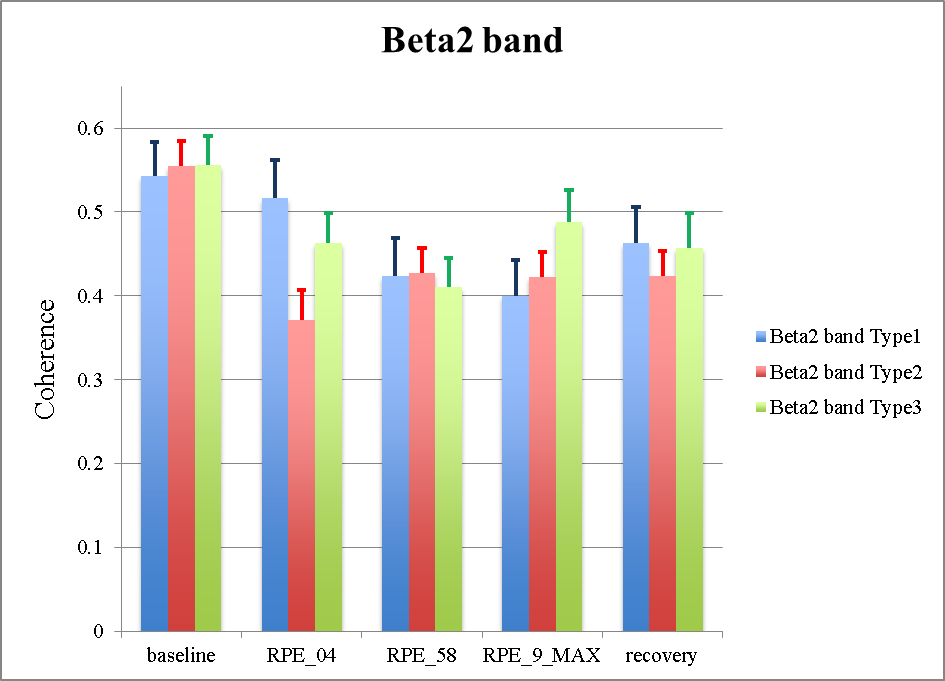


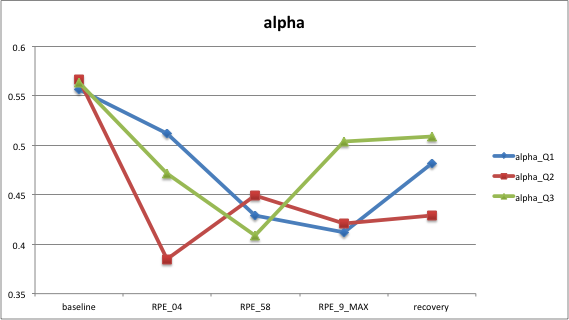


Figure 4S. Averaged coherence values (not tresholded) of the 10 selected electrodes (F3, F4, C3, C4, P3, P4, T7, T8, O1, O2) for each Type of performance during each effort period in beta 3 band


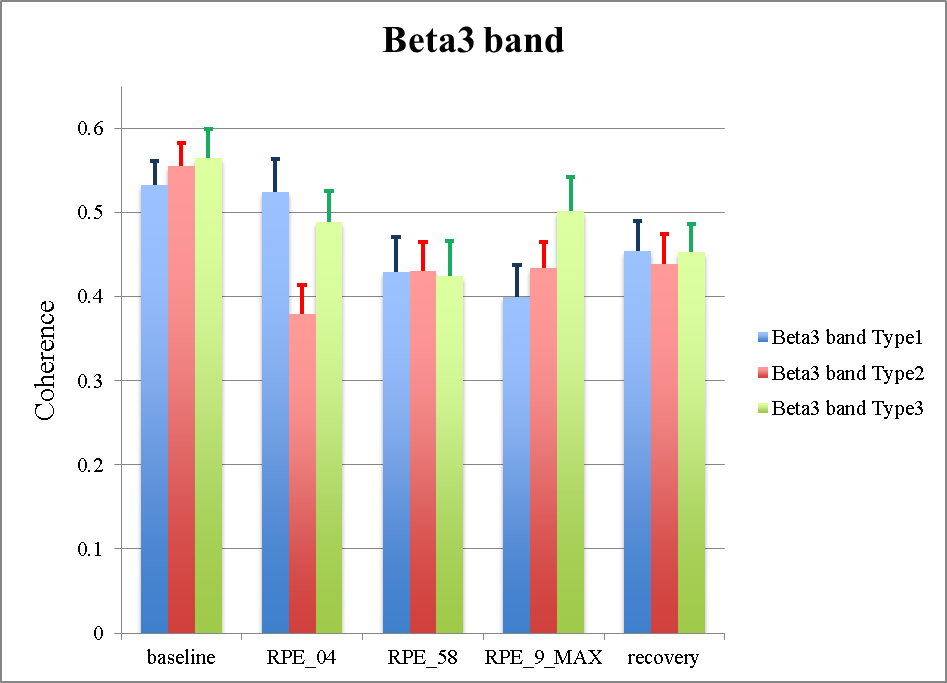


*Table 1S.* RM-ANOVA 3 (performance) × 5 (effort level) results for the alpha band using Greenhouse Geisser correction (**p* < 0.05)

| Electrode Pairs | | Factors | df | F | *p* | η_p_^2^ | Power |
| --- | --- | --- | --- | --- | --- | --- | --- |
| F3-F4 | Performance | | 1,67 | 1.69 | 0.215 | 0.145 | 0.284 |
| F3-P3 |  | | 1,92 | 0.48 | 0.614 | 0.047 | 0.117 |
| F4-P4 |  | | 1,74 | 0.34 | 0.068 | 0.033 | 0.094 |
| T7-T8 |  | | 1,98 | 0.11 | 0.894 | 0.011 | 0.065 |
| T7-P3 |  | | 1,59 | 1.73 | 0.209 | 0.148 | 0.283 |
| C3-C4 |  | | 1,89 | 1.72 | 0.206 | 0.147 | 0.309 |
| C3-P3 |  | | 3,52 | 1.36 | 0.211 | 0.135 | 0.405 |
| C4-P4 |  | | 1,43 | 0.68 | 0.473 | 0.064 | 0.132 |
| T8-P4 |  | | 1,25 | 0.04 | 0.879 | 0.005 | 0.055 |
| P3-P4 |  | | 1,73 | 0.03 | 0.965 | 0.004 | 0.055 |
| P3-O1 |  | | 1,98 | 0.04 | 0.955 | 0.005 | 0.056 |
| P4-O2 |  | | 1,71 | 0.89 | 0.412 | 0.082 | 0.171 |
| O1-O2 |  | | 1,62 | 1.39 | 0.271 | 0.123 | 0.238 |
| F3-F4 | Effort lev | | 3,02 | 3.17 | 0.038* | 0.241 | 0.678 |
| F3-P3 |  | | 3,14 | 4.57 | 0.008* | 0.314 | 0.855 |
| F4-P4 |  | | 2,47 | 4.97 | 0.011* | 0.332 | 0.816 |
| T7-T8 |  | | 2,51 | 3.83 | 0.027* | 0.277 | 0.706 |
| T7-P3 |  | | 2,19 | 4.55 | 0.020* | 0.313 | 0.738 |
| C3-C4 |  | | 3,36 | 3.74 | 0.017* | 0.272 | 0.792 |
| C3-P3 |  | | 2,58 | 4.81 | 0.011* | 0.325 | 0.816 |
| C4-P4 |  | | 2,61 | 6.51 | 0.003* | 0.394 | 0.924 |
| T8-P4 |  | | 2,88 | 3.15 | 0.041* | 0.240 | 0.659 |
| P3-P4 |  | | 2,87 | 5.43 | 0.005* | 0.352 | 0.893 |
| P3-O1 |  | | 2,92 | 2.66 | 0.068 | 0.210 | 0.582 |
| P4-O2 |  | | 2,68 | 4.80 | 0.010* | 0.325 | 0.820 |
| O1-O2 |  | | 3,03 | 2.61 | 0.068 | 0.207 | 0.586 |
| F3-F4 | Perf × Effort lev | | 4,28 | 0.82 | 0.523 | 0.076 | 0.358 |
| F3-P3 |  | | 3,01 | 1.12 | 0.353 | 0.101 | 0.274 |
| F4-P4 |  | | 4,32 | 1.37 | 0.258 | 0.332 | 0.816 |
| T7-T8 |  | | 3,60 | 1.14 | 0.347 | 0.103 | 0.308 |
| T7-P3 |  | | 4,20 | 1.84 | 0.135 | 0.156 | 0.527 |
| C3-C4 |  | | 3,53 | 1.92 | 0.135 | 0.161 | 0.492 |
| C3-P3 |  | | 3,52 | 1.36 | 0.211 | 0.135 | 0.405 |
| C4-P4 |  | | 4,47 | 0.88 | 0.491 | 0.081 | 0.272 |
| T8-P4 |  | | 4,76 | 1.24 | 0.303 | 0.111 | 0.393 |
| P3-P4 |  | | 4,28 | 1.04 | 0.403 | 0.094 | 0.308 |
| P3-O1 |  | | 3,38 | 1.64 | 0.193 | 0.141 | 0.415 |
| P4-O2 |  | | 4,38 | 1.07 | 0.382 | 0.097 | 0.325 |
| O1-O2 |  | | 4,52 | 1.38 | 0.251 | 0.122 | 0.421 |

*Table 2S*. RM-ANOVA 3 (performance) × 5 (effort level) results for the beta1 band using Greenhouse Geisser correction (**p* < 0.05)

| Electrode Pairs | | Factors | df | F | *p* | η_p_^2^ | power |
| --- | --- | --- | --- | --- | --- | --- | --- |
| F3-F4 | Performance | | 1,85 | 0.42 | 0.645 | 0.041 | 0.107 |
| F3-P3 |  | | 1,57 | 0.27 | 0.714 | 0.026 | 0.083 |
| F4-P4 |  | | 1,90 | 0.84 | 0.441 | 0.078 | 0.170 |
| T7-T8 |  | | 1,92 | 0.01 | 0.985 | 0.001 | 0.052 |
| T7-P3 |  | | 1,51 | 0.43 | 0.602 | 0.041 | 0.102 |
| C3-C4 |  | | 1,67 | 1.39 | 0.273 | 0.122 | 0.240 |
| C3-P3 |  | | 1,77 | 2.50 | 0.110 | 0.204 | 0.422 |
| C4-P4 |  | | 1,47 | 0.09 | 0.850 | 0.010 | 0.061 |
| T8-P4 |  | | 1,51 | 0.07 | 0.885 | 0.007 | 0.058 |
| P3-P4 |  | | 1,85 | 0.11 | 0.880 | 0.011 | 0.064 |
| P3-O1 |  | | 1,39 | 0.11 | 0.829 | 0.011 | 0.062 |
| P4-O2 |  | | 1,58 | 1.40 | 0.270 | 0.123 | 0.235 |
| O1-O2 |  | | 1,57 | 1.05 | 0.356 | 0.095 | 0.186 |
| F3-F4 | Effort lev | | 2,92 | 3.78 | 0.021* | 0.275 | 0.752 |
| F3-P3 |  | | 3,08 | 4.87 | 0.006* | 0.328 | 0.873 |
| F4-P4 |  | | 2.32 | 3.24 | 0.050* | 0.245 | 0.600 |
| T7-T8 |  | | 2,45 | 1.60 | 0.218 | 0.138 | 0.336 |
| T7-P3 |  | | 2,78 | 3.39 | 0.034* | 0.254 | 0.682 |
| C3-C4 |  | | 2,47 | 2.67 | 0.115 | 0.185 | 0.461 |
| C3-T3 |  | | 2,25 | 5.10 | 0.012* | 0.338 | 0.798 |
| C4-P4 |  | | 2,76 | 5.77 | 0.004* | 0.366 | 0.902 |
| T8-P4 |  | | 2,91 | 2.68 | 0.060 | 0.212 | 0.585 |
| P3-P4 |  | | 2,54 | 4.85 | 0.011* | 0.327 | 0.816 |
| P3-O1 |  | | 2,91 | 2.96 | 0.050* | 0.228 | 0.631 |
| P4-O2 |  | | 2,47 | 3.95 | 0.025* | 0.283 | 0.713 |
| O1-O2 |  | | 3,21 | 3.98 | 0.014* | 0.285 | 0.805 |
| F3-F4 | Perf × Effort lev | | 3,20 | 1.06 | 0.380 | 0.097 | 0.274 |
| F3-P3 |  | | 3,62 | 0.90 | 0.418 | 0.090 | 0.269 |
| F4-P4 |  | | 3,77 | 1.54 | 0.211 | 0.134 | 0.419 |
| T7-T8 |  | | 4,86 | 1.25 | 0.300 | 0.111 | 0.400 |
| T7-P3 |  | | 4,61 | 1.29 | 0.285 | 0.114 | 0.339 |
| C3-C4 |  | | 4,56 | 1.56 | 0.193 | 0.135 | 0.476 |
| C3-P3 |  | | 4,40 | 1.03 | 0.403 | 0.094 | 0.314 |
| C4-P4 |  | | 4,15 | 1.72 | 0.160 | 0.147 | 0.492 |
| T8-P4 |  | | 3,96 | 2.34 | 0.072 | 0.190 | 0.623 |
| P3-P4 |  | | 4,51 | 0.85 | 0.514 | 0.078 | 0.262 |
| P3-O1 |  | | 3,61 | 1.55 | 0.212 | 0.134 | 0.409 |
| P4-O2 |  | | 4,98 | 1.61 | 0.175 | 0.139 | 0.514 |
| O1-O2 |  | | 4,63 | 1.02 | 0.409 | 0.093 | 0.321 |

*Table 3S*. RM-ANOVA 3 (performance) × 5 (effort level) results for the beta 2 band using Greenhouse Geisser correction (**p* < 0.05)

| Electrode Pairs | | Factors | df | F | *p* | η_p_^2^ | power |
| --- | --- | --- | --- | --- | --- | --- | --- |
| F3-F4 | Performance | | 1,63 | 2.74 | 0.102 | 0.215 | 0.426 |
| F3-P3 |  | | 1,76 | 3.33 | 0.064 | 0.250 | 0.524 |
| F4-P4 |  | | 1,97 | 0.40 | 0.668 | 0.039 | 0.106 |
| T7-T8 |  | | 1,73 | 0.19 | 0.801 | 0.018 | 0.073 |
| T7-P3 |  | | 1,13 | 0.19 | 0.801 | 0.018 | 0.073 |
| C3-C4 |  | | 1,69 | 2.17 | 0.150 | 0.179 | 0.355 |
| C3-P3 |  | | 1,94 | 2.19 | 0.139 | 0.180 | 0.388 |
| C4-P4 |  | | 1,65 | 0.89 | 0.408 | 0.083 | 0.168 |
| T8-P4 |  | | 1,54 | 0.04 | 0.918 | 0.005 | 0.056 |
| P3-P4 |  | | 1,75 | 0.01 | 0.975 | 0.002 | 0.052 |
| P3-O1 |  | | 1,72 | 0.76 | 0.463 | 0.071 | 0.152 |
| P4-O2 |  | | 1,34 | 0.70 | 0.458 | 0.066 | 0.131 |
| O1-O2 |  | | 1,50 | 1.15 | 0.320 | 0.104 | 0.196 |
| F3-F4 | Effort lev | | 2,57 | 4.95 | 0.010* | 0.331 | 0.828 |
| F3-P3 |  | | 2,72 | 6.36 | 0.003* | 0.389 | 0.926 |
| F4-P4 |  | | 2,08 | 4.17 | 0.029* | 0.294 | 0.68 |
| T7-T8 |  | | 2,52 | 2.25 | 0.116 | 0.184 | 0.463 |
| T7-P3 |  | | 2,61 | 2.94 | 0.058 | 0.227 | 0.592 |
| C3-C4 |  | | 2,59 | 5.76 | 0.005* | 0.336 | 0.886 |
| C3-T3 |  | | 2,51 | 5.94 | 0.005* | 0.373 | 0.888 |
| C4-P4 |  | | 2,53 | 4.70 | 0.013* | 0.320 | 0.800 |
| T8-P4 |  | | 2,60 | 4.32 | 0.017* | 0.302 | 0.773 |
| P3-P4 |  | | 2,88 | 4.31 | 0.013* | 0.301 | 0.805 |
| P3-O1 |  | | 3,21 | 3.58 | 0.022* | 0.264 | 0.758 |
| P4-O2 |  | | 2,72 | 3.11 | 0.047* | 0.238 | 0.634 |
| O1-O2 |  | | 3,23 | 2.66 | 0.061 | 0.210 | 0.616 |
| F3-F4 | Perf × Effort lev | | 4,12 | 1.04 | 0.398 | 0.094 | 0.304 |
| F3-P3 |  | | 4,36 | 2.18 | 0.081 | 0.179 | 0.62 |
| F4-P4 |  | | 3,19 | 1.67 | 0.191 | 0.143 | 0.407 |
| T7-T8 |  | | 4,69 | 1.43 | 0.232 | 0.125 | 0.445 |
| T7-P3 |  | | 3,17 | 1.30 | 0.289 | 0.116 | 0.323 |
| C3-C4 |  | | 4,53 | 1.56 | 0.194 | 0.135 | 0.473 |
| C3-P3 |  | | 3,99 | 1.02 | 0.406 | 0.093 | 0.293 |
| C4-P4 |  | | 4,54 | 2.45 | 0.063 | 0.197 | 0.686 |
| T8-P4 |  | | 5,02 | 1.51 | 0.347 | 0.103 | 0.375 |
| P3-P4 |  | | 4,4 | 0.43 | 0.799 | 0.042 | 0.147 |
| P3-O1 |  | | 4,00 | 0.51 | 0.724 | 0.049 | 0.160 |
| P4-O2 |  | | 4,43 | 1.91 | 0.118 | 0.161 | 0.561 |
| O1-O2 |  | | 4,28 | 1.22 | 0.310 | 0.109 | 0.364 |

*Table 4S*. RM-ANOVA 3 (performance) × 5 (effort level) results in beta 3 band using Greenhouse Geisser correction (**p* < 0.05)

| Electrode Pairs | Factors | df | F | *p* | η_p_^2^ | power |
| --- | --- | --- | --- | --- | --- | --- |
| F3-F4 | Performance | 1,6 | 1.86 | 0.191 | 0.157 | 0.301 |
| F3-P3 |  | 1,9 | 2.25 | 0.134 | 0.184 | 0.393 |
| F4-P4 |  | 1,98 | 0.56 | 0.578 | 0.053 | 0.130 |
| T7-T8 |  | 1,98 | 0.20 | 0.815 | 0.020 | 0.077 |
| T7-P3 |  | 1,14 | 0.75 | 0.421 | 0.070 | 0.129 |
| C3-C4 |  | 1,87 | 0.70 | 0.499 | 0.066 | 0.148 |
| C3-P3 |  | 1,94 | 1.98 | 0.165 | 0.166 | 0.356 |
| C4-P4 |  | 1,56 | 0.142 | 0.819 | 0.014 | 0.067 |
| T8-P4 |  | 1,60 | 0.090 | 0.869 | 0.010 | 0.061 |
| P3-P4 |  | 1,52 | 0.91 | 0.396 | 0.084 | 0.165 |
| P3-O1 |  | 1,83 | 1.47 | 0.254 | 0.128 | 0.264 |
| P4-O2 |  | 1,59 | 0.63 | 0.508 | 0.060 | 0.130 |
| O1-O2 |  | 1,31 | 0.88 | 0.393 | 0.081 | 0.152 |
| F3-F4 | Effort lev | 2,81 | 4.85 | 0.009* | 0.327 | 0.486 |
| F3-P3 |  | 2,76 | 11.30 | 0.001* | 0.531 | 0.997 |
| F4-P4 |  | 2,19 | 2.64 | 0.089 | 0.209 | 0.491 |
| T7-T8 |  | 2,64 | 3.11 | 0.048* | 0.238 | 0.624 |
| T7-P3 |  | 2,80 | 3.35 | 0.036* | 0.251 | 0.678 |
| C3-C4 |  | 2,24 | 4.07 | 0.027* | 0.289 | 0.694 |
| C3-P3 |  | 1,87 | 3.83 | 0.043* | 0.277 | 0.605 |
| C4-P4 |  | 2,61 | 6.02 | 0.004* | 0.376 | 0.901 |
| T8-P4 |  | 1,87 | 4.44 | 0.028* | 0.308 | 0.674 |
| P3-P4 |  | 2,13 | 3.04 | 0.066 | 0.233 | 0.544 |
| P3-O1 |  | 2,45 | 2.88 | 0.066 | 0.224 | 0.562 |
| P4-O2 |  | 2,50 | 3.88 | 0.026* | 0.280 | 0.710 |
| O1-O2 |  | 2,77 | 3.97 | 0.020* | 0.285 | 0.757 |
| F3-F4 | Perf × Effort lev | 4,01 | 1.14 | 0.352 | 0.102 | 0.325 |
| F3-P3 |  | 4,29 | 1.72 | 0.158 | 0.147 | 0.501 |
| F4-P4 |  | 3,65 | 0.99 | 0.415 | 0.091 | 0.273 |
| T7-T8 |  | 4,81 | 1.18 | 0.330 | 0.106 | 0.377 |
| T7-P3 |  | 3,84 | 0.91 | 0.462 | 0.084 | 0.258 |
| C3-C4 |  | 4,77 | 2.62 | 0.038* | 0.208 | 0.742 |
| C3-P3 |  | 3,32 | 1.24 | 0.311 | 0.110 | 0.316 |
| C4-P4 |  | 4,81 | 1.89 | 0.115 | 0.159 | 0.582 |
| T8-P4 |  | 3,67 | 1.75 | 0.164 | 0.149 | 0.463 |
| P3-P4 |  | 4,55 | 0.65 | 0.649 | 0.061 | 0.207 |
| P3-O1 |  | 4,83 | 1.07 | 0.387 | 0.097 | 0.342 |
| P4-O2 |  | 4,72 | 2.70 | 0.064 | 0.213 | 0.754 |
| O1-O2 |  | 3,86 | 1.13 | 0.356 | 0.102 | 0.316 |
